# Interoceptive Abilities in Inflammatory Bowel Diseases and Irritable Bowel Syndrome

**DOI:** 10.3389/fpsyt.2020.00229

**Published:** 2020-04-02

**Authors:** Alicia Fournier, Laurie Mondillon, Olivier Luminet, Fréderic Canini, Nicolas Mathieu, Anne Sophie Gauchez, Cécile Dantzer, Bruno Bonaz, Sonia Pellissier

**Affiliations:** ^1^ Université de Bourgogne, Laboratoire Psy-DREPI, Dijon, France; ^2^ MSHE Claude-Nicolas Ledoux, USR3124, Behaviors, Risk and Health, Besançon, France; ^3^ Université Clermont Auvergne, CNRS, Laboratoire de Psychologie Sociale et Cognitive, Team on Physiological and Psychosocial Stress, Well-being Physiological and Psychosocial Stress, Clermont-Ferrand, France; ^4^ Research Institute for Psychological Sciences, Université catholique de Louvain, Louvain-La-Neuve, Belgium; ^5^ Fund for Scientific Research (FRS-FNRS), Brussels, Belgium; ^6^ Unité de Neurophysiologie du Stress, Institut de Recherche Biomédicale des Armées (IRBA), Brétigny sur Orge, France; ^7^ École du Val de Grâce, Paris, France; ^8^ Service d’Hépato-Gastroentérology, Centre Hospitalier Universitaire de Grenoble, Grenoble, France; ^9^ Institut de Biologie et Pathologie, Centre Hospitalier Universitaire de Grenoble, Grenoble, France; ^10^ Université de Bordeaux, Laboratoire de Psychologie, Bordeaux, France; ^11^ Université Grenoble Alpes, Grenoble Institute of Neurosciences, Grenoble, France; ^12^ Université Savoie Mont Blanc, LIP/PC2S, Chambéry, France

**Keywords:** inflammatory bowel disease, irritable bowel syndrome, alexithymia, interoceptive abilities, hypothalamic-pituitary adrenal axis

## Abstract

Alexithymia is usually described by three main dimensions difficulty identifying feelings (DIF), difficulty describing feelings (DDF), and externally oriented thinking (EOT). The most commonly used questionnaire investigating alexithymia, the Toronto Alexithymia Scale (TAS-20), supports this three-factor structure. One important assumption is that alexithymia severity is associated to vulnerability to somatic diseases, among them gastrointestinal disorders. However, the association between alexithymia and gastrointestinal disorders is not systematic, thus questioning the role of alexithymia as a vulnerability factor for those illnesses. A recent factor analysis suggested another four-factor structure for the TAS-20: difficulties in awareness of feelings (DAF), difficulties in interoceptive abilities (DIA), externally oriented thinking (EOT), and poor affective sharing (PAS). We assume that DIA and DAF might be more relevant to investigate the association between alexithymia and gastrointestinal disorders. The rationale is that DIA and DAF reflect impairments in emotion regulation that could contribute to an inappropriate autonomic and HPA axis homeostasis in irritable bowel syndrome (IBS), ulcerative colitis (UC), or Crohn’s disease (CD). The aim of this study was to investigate whether DIA and DAF are associated with the presence of IBS, UC or CD, while checking for anxiety, depression, parasympathetic (vagus nerve) activity and cortisol levels. We recruited control participants (n=26), and patients in remission who were diagnosed with IBS (n=24), UC (n=18), or CD (n=21). Participants completed questionnaires to assess anxiety, depression, and alexithymia. A blood sample and an electrocardiogram were used to measure the level of cortisol and parasympathetic activity, respectively. Logistic regressions with the four-factor structure of the TAS-20 revealed that DIA was a significant predictor of IBS (*W*(1)=6.27, *p*=.01). Conversely, DIA and DAF were not significant predictors in CD and UC patients. However, low cortisol level was a significant predictor of UC (*W*(1)=4.67, *p*=.035). Additional logistic regressions based on the original 3-factor structure of TAS-20 (DIF, DDF, and EOT) showed that only DDF was a significant predictor of CD [*W*(1)=6.16, *p* < .001]. The present study suggests that DIA is an important dimension for assessing potential risk for gastrointestinal diseases, in particular for IBS.

## Introduction

Among gastrointestinal pathologies, the most common diagnoses in gastroenterology are irritable bowel syndrome (IBS) and inflammatory bowel diseases (IBD). Although these are common pathologies that affect the intestine and are characterised by alternating periods of remission and relapse, only the intestinal symptoms (e.g., abdominal pain and chronic diarrhoea) are similar. The main difference is that IBS is a functional disorder (i.e., without structural abnormalities) (1) while IBD are structural diseases where digestive and extra-digestive manifestations are caused by chronic inflammation (2). IBD include ulcerative colitis (UC) and Crohn’s disease (CD). Patients with UC have inflammation confined to the rectum and colon, while patients with CD have inflammation that can affect the entire gastrointestinal tract.

Although the aetiology is multifactorial, numerous studies have agreed that among environmental factors, stress has a major impact on the development and/or relapse of IBS and, more recently, IBD. This is due to the existence of bidirectional communication between the brain and the gut through the autonomic nervous system (ANS), the hypothalamo-pituitary-adrenal (HPA) axis, and the gastrointestinal tract (gastrointestinal immune system, intestinal barrier, enteric nervous system and microbiota) (i.e., brain-gut-microbiota axis) (3, 4). Another explanation for the relapse of functional gastrointestinal disorders is the important role of psychological status and physiological stimuli (5). In IBS and IBD patients, the presence of adverse symptoms, anxiety, depression (6, 7), as well as individual visceral hypersensitivity (8) is critical for the regulation of psychological mood, which in turn may influence the perception and generation of regulatory physiological signals (9).

Depending on the studies, IBS patients have an increase of sympathetic activity at rest (10) or under stress (11), or a decrease of vagal tone (12, 13). Moreover, IBS patients with low vagal tone are reported to have high levels of blood epinephrine at rest (14). Among IBD patients, UC patients exhibit high sympathetic (15–17) and low vagal tone related to negative affects compared to healthy individuals (18). CD patients also present higher sympathetic-parasympathetic ratio compared to control individuals, which can be explained by an increase in sympathetic activity (19). However, the overactivity of the HPA axis in patients with gastrointestinal pathologies remains controversial. IBS patients facing a psychological stressor exhibit an overactivity of the HPA axis (20, 21). Compared to healthy individuals, IBS patients at rest have salivary cortisol levels higher in the morning and lower in the evening (22), a difference not always observed (23) except in patients with low vagal tone (14). Conversely, IBD patients exhibit hypocortisolism rather than a hypercortisolism profile (24, 25). The communication between the brain and the gastrointestinal tract (i.e., brain-gut-microbiota axis) is mediated by the HPA axis, the ANS and the immune system. As a result, alterations in the functioning of these systems can lead to impaired motor, sensory, secretory, and immune functions of the gastrointestinal tract, the development of inflammatory-related or immunosuppressed medical conditions, leading to depression and anxiety that could accelerate a relapse (4).

Besides differences in stress mediator regulation, IBS and IBD also present an impairment in interoception (26, 27) and high levels of alexithymia (28, 29). Interoception refers to the ability to accurately detect internal body changes. Interoceptive abilities are composed of different facets: (i) interoceptive accuracy (objective ability to perceive internal body changes), (ii) interoceptive sensibility (subjective ability to report on body states), (iii) interoceptive awareness (degree of overlap between interoceptive accuracy and sensibility), and (iv) interoceptive emotional evaluation (emotional degree attributed to body sensations that are expressed or paid attention to in a specific situation) (30). Interoception is considered a necessary component of the emotional experience (31, 32). It implies that the evaluation and perception of one’s own emotional states cannot be separated from the processes of evaluating one’s own body experiences (33). This connection is illustrated by a majority of studies looking at self-reported interoception that find a link with alexithymia (34–39). Alexithymia is a personality construct that implies difficulties identifying and describing one’s own feelings, limited imaginative processes, an externally oriented cognitive style, and difficulties in distinguishing between feelings and bodily sensations (40–42). This construct is associated with many disorders, such as gastrointestinal pathologies (28, 29). This association can be explained by deficits in the strategies used in emotion regulation (43). In this perspective, a recent study suggests that only the difficulties identifying and describing one’s own feelings and an externally oriented cognitive style dimensions define the alexithymia construct. Difficulties identifying and describing one’s own feelings dimensions would correspond to difficulties in the *appraisal* stage of emotion valuation system and externally oriented cognitive style dimension would correspond to difficulties in the *attention* stage of emotion valuation system (44). Emotion regulation involves mechanisms that allow individuals to modulate their emotions during environmental demands (45). This ability is an essential component for the general well-being of individuals. If the emotion regulation mechanisms are not sufficiently efficient, individuals are more vulnerable to developing a chronic stress state and in the long term psychological and somatic diseases (46). For example, an individual with difficulties in detecting, processing or becoming aware of internal body changes (interoception) is also likely to have difficulties identifying and describing feelings (alexithymia). When faced with challenging emotional situations, he/she could exhibit difficulties regulating his/her emotions and autonomous functioning.

In patients in remission, we tested a vulnerability model in which difficulties in interoceptive abilities (DIA) and in awareness of feelings (DAF) could potentially represent vulnerability factors, while checking for psychological (anxiety, depression) and physiological (activity of the parasympathetic ANS and the HPA axis) factors. We hypothesize that DIA and DAF could predict the presence of IBS or IBD (both CD and UC). We focused on DIA and DAF because these dimensions could explain difficulties in emotion regulation and, consequently, explain physiological and psychological alterations that could contribute to an increase in and/or inappropriate stress response and the recurrence or aggravation of gastrointestinal pathologies. Although the EOT dimension appears to assess difficulties in emotion regulation (44), we decided not to use this dimension for two reasons. Firstly, its internal reliability is usually poor (47–50). Secondly, the dimension might better reflect the social norms that guide emotional behaviors rather than a cognitive style of thinking (51). Identifying factors that affect the well-being of patients with IBS or IBD in remission renders possible an intervention to modify some of these factors or promote a better understanding of patients with IBS or IBD who experience alternate periods of remission and relapse.

## Method

### Participants and Ethics Statement

Patients in remission and clinically diagnosed with IBS (mean age: 38.21 ± 11.26 years; 7 men, 17 women), UC (mean age: 40.94 ± 10.78 years; 9 men, 9 women), or CD (mean age: 40.29 ± 11.17 years; 9 men, 12 women) were recruited from the Gastroenterology Department of Grenoble University Hospital (CHU) between September 2009 and October 2011. Patients with IBS were selected according to the Rome II criteria (52). Patients with UC and CD were recruited according to their pathology activity index, respectively UC activity index (UCAI) (53) and Harvey-Bradshaw index (HBI) (54). UC patients with a UCAI ≤ 10 and CD patients with an HBI < 4 on inclusion were considered in clinical remission. In addition, we recruited healthy control (HC) participants (mean age: 36.23 ± 10.06 years; 8 men, 18 women), with no chronic medication intake for any disease, from a list of healthy volunteers from the Grenoble INSERM Clinical Investigation Centre.

The study was conducted according to the Declaration of Helsinki and in accordance with the guidelines of Good Clinical Practice. The Ethical Committee of the CHU approved the study (ref: CPP 08-CHUG-23; ClinicalTrials.gov Identifier: NCT010950421). Some of the results of this protocol have already been published twice (14, 29).

### Criteria for Exclusion

Patients were excluded from the study if they (*i*) had past or present severe disorders (e.g., diabetes, heart failure, dysthyroidism, renal or liver insufficiency, malignant condition, alcoholism, psychiatric disorders, and amyloidosis); (*ii*) had autonomic dysfunction (e.g., peripheral neuropathy, vagotomy, and asthma); (*iii*) were taking medication that could alter ANS functioning (e.g., anticholinergics, antiarrhythmics, clonidine, and β-blocking agents); (*iv*) were pregnant or breast-feeding; and/or (*v*) had past abdominal surgery except for appendectomy and/or cholecystectomy.

### Materials

#### Psychological Assessments

Interoceptive abilities and awareness of feelings were assessed using a part ofthe French version of the Toronto Alexithymia Scale (TAS-20) (55, 56). The TAS-20 allows the assessment of three dimensions: difficulty identifying feelings (DIF; items 1, 3, 6, 7, 9, 13, 14; α=.83), difficulty describing feelings (DDF; items 2, 4, 11, 12, 17; α=.65), and EOT (items 5, 8, 10, 15, 16, 18, 19, 20; α=.60). However, based on literature highlighting interoceptive alteration in alexithymic individuals, Fournier and colleagues (2019) propose a new structure for this scale without items 16 and 20. This new structure is composed of four dimensions: DIA (tems 1, 2, 4, 6, 9, 11, 13, 14; α=.81), difficulty in interoceptive abilities (DIA; items 3^1^, 7^2^; α=.77), externally oriented thinking (EOT; items 5, 8, 10, 18, 19; α=.48), and poor affective sharing (PAS; items 4, 11, 12, 15, 17; α=.63). For this study, we decided to use the factor structure proposed by Fournier and colleagues, because the DIA and DAF dimensions were predominantly associated with a deficit in emotion regulation (i.e., depression, anxiety, emotional instability, high level of perceived stress, use of dysfunctional coping strategies) and DIA dimension was specifically associated with health disorders (i.e., somatic disorders, eating disorders, medication intake, cardiovascular disease) (57). A high score of DIA indicates a high level of DIA and a high score of DAF indicates a high level of difficulty in awareness of feelings.

Depressive symptomatology was assessed using the French version of the Center for Epidemiologic Studies–Depression scale (CES-D) (58, 59). This brief scale of 20 items assesses symptoms and behaviors often associated with depression. A high score means a high level of depressive symptomatology (α=.87).

Anxiety trait was assessed using the French version of the State–Trait Anxiety Inventory (STAI-Y) (60, 61). This scale consists of 20 items. A high score indicates high anxiety (α=.91).

#### HRV for Parasympathetic Activity Assessment

Parasympathetic activity was evaluated by investigating heart rate variability. We performed an electrocardiogram (ECG) using electrodes placed on each wrist. Heart rate variability analysis was performed using the Heart Rhythm Scanner software (Biocom Technologies, USA). QRS complexes were automatically classified, and then visually checked to detect and remove abnormal complexes. A standard spectral analysis was applied to inter-beat intervals using a Fast Fourier Transformation (FFT) according to the standards of measurement of the task force on heart rate variability (62). We considered the high frequency (HF) spectrum (from 0.15 to 0.40 Hz.ms^2^) that reflects 90% parasympathetic tone fluctuations caused by respiratory sinus arrhythmia at rest in a sitting position (63). We computed normalized scores [HFnu = HF/(TP–VLF)] to minimise the effect of changes in very low frequency power on HF power and emphasised the changes in parasympathetic regulation.

#### Blood Samples for Neuroendocrine Assessment

Blood samples were collected between 8:30 am and 9:00 am. Cortisol levels were measured in serum by means of a competitive immunoassay using direct chemiluminescent technology on ADVIA Centaur^®^ XP (Siemens Health Care Diagnostics, Saint Denis, France).

### Procedure

Upon arrival in the Gastroenterology Department of Grenoble University Hospital, participants were welcomed in a quiet room. The experimenter verified the inclusion/exclusion conditions in each group. The nature and potential risks of the study were fully explained, and written informed consent was obtained from each participant.

After this short interview, the participant started the experimental protocol. All sessions took place between 8:00 am to 10:00 am, after a light breakfast to avoid strong influences of circadian and postprandial variations. At the beginning of the protocol, participants completed psychological questionnaires (i.e., STAI-Y, CES-D, TAS-20). Then, a nurse placed electrodes on the wrists (ECG recording) and an intravenous catheter in the arm vein (blood sample). A standardized interview with questions mainly related to the symptomatology and history of the pathology) was then carried out for 30 min. This interview also allowed participants to recover from the stress of the prick. After this resting period, participants rated the intensity of their current visceral pain using a visual analogue scale (0: no perceived pain; 10: maximum perceived pain). Finally, a 10-min ECG was recorded, and a 30-ml blood sample was taken immediately after the ECG recording. The overall study design consisted of two parts. The analyses presented here concern only the first part of the study [for a complete description of the study see Fournier and colleagues (29)].

### Statistical Analysis

Statistical analyses were carried out with SPSS v.24.0 (IBM Corp., USA). Data are expressed as means (± standard deviation, SD). The *p-*value for statistical significance was set at *p* ≤.05, and the trend for significance was set at *p* ≤ .07.

ANOVAs were used to evaluate the main effects of groups on visceral pain, and psychological, biological, and physiological measures. When a significant effect was observed, a Bonferroni *post hoc* test was applied to determine the differences between each group. If the homoscedasticity was not respected, we used adjusted *Welch’s F*. For two-by-two comparisons, we used a Bonferroni *post hoc* test to determine the differences between each group.

Then, to examine the predictive value of the DAF and DIA dimensions on the presence of gastrointestinal pathologies, we performed hierarchical logistic regressions with the patient group as the dependent variable (considering separately IBS, UC, and CD). The presence of gastrointestinal pathologies was coded 1 and the absence of disorder (HC group) was coded 0. It is known that anxiety, depression and cortisol levels are associated with gastrointestinal disorders (64). In addition, autonomic dysfunction can be a confused factor between digestive disorders and alexithymia. In order to examine the specific contribution of DAF and DIA dimensions and avoid cooccurrences, we checked for any possible effects of anxiety and depression by entering STAI-Y and CES-D at Step 1. Then, we checked cortisol levels and autonomic activity by entering cortisol and HFnu at Step 2 and we entered our dimensions of interest, DAF and DIA dimensions, at Step 3. A significant improvement of the *χ^2^* (observed from the Δ*χ* ^2^=*χ^2^*_new model_-*χ^2^*_previous model_) means that the new model is better than the previous model. Despite the fact that the DIA dimension had good internal reliability, this dimension includes only two items (57). For this reason, we carried out additional analyses by including, instead of DAF and DIA dimensions, the theoretical DIF and DDF dimensions of the TAS-20 as predictors. We decided to keep only these two dimensions because they included all the items distributed in the DAF and DIA dimensions.

Given the restricted sample size, we used bootstrapping to perform hierarchical logistic regressions. The level of confidence was 95% and we used a bootstrap percentile method for 5,000 bootstrap replications. The odds ratio was used to compare the relative odds of the occurrence of the outcome of interest (i.e., IBS, UC, CD), given exposure to the variable of interest (i.e., anxiety, depression, HFnu, cortisol, DAF/DIF, DIA/DDF). An odds ratio equal to one means that exposure to the variable of interest (anxiety, depression, HFnu, cortisol, DAF/DIF, or DIA/DDF) does not affect the odds of the occurrence of a gastrointestinal pathology (IBS, UC, or CD); an odds ratio greater than one means that exposure to the variable of interest is associated with higher odds of the occurrence of a gastrointestinal pathology; and an odds ratio less than one means that exposure to the variable of interest is associated with lower odds of the occurrence of a gastrointestinal pathology (65). An absence of multicollinearity between predictor variables was verified before performing hierarchical logistic regressions. Only results with a confidence interval not including zero were retained and interpreted.

## Results

Differences between groups. The groups did not differ in age or on EOT, PAS scores of the TAS without items 16 and 20, EOT of the TAS-20 and HFnu index (*ps* > .07*)*. However, there was a significant effect of the disease on the level of perceived visceral pain [*Welch F*(3,43.82)=5.11, *p*=.004]. IBS had the highest score of perceived visceral pain compared to controls (*p*=.001). There was also a significant effect of the disease on the scores of anxiety (*F*(3, 85)=6.69, *p* < .001)] and depressive symptomatology [*Welch F*(3,42.88)=9.75, *p* < .001]. IBS and CD patients had higher scores of anxiety than HC participants (*p* < .001 and *p*=.021 respectively), and IBS patients had the highest scores of depressive symptomatology in comparison to HC participants and CD and UC patients (*p* < .001, *p*=.031, *p* < .001 respectively).

There was a significant effect of the disease on the total alexithymia (TAS-20 without items 16 and 20) scores [*F*(3, 78)=4.02, *p*=.01], DAF scores [*F*(3, 83)=3.43, *p*=.021], DIA scores [*Welch F*(3,41.63)=9.18, *p* < .001], PAS scores [*Welch F*(3,41.71)=3.54, *p*=.023], on the total TAS-20 scores [*F*(3, 77)=3.56, *p*=.018], DIF scores [*Welch F*(3,45.14)=4.93, *p*=.005)] and DDF scores [*Welch F*(3,44.09)=3.14, *p*=.035]. CD patients were more alexithymic overall (TAS-20 without items 16 and 20: *p*=.018; TAS-20: *p*=.041) and had more difficulties in awareness of feelings, in interoceptive abilities and in identifying feelings than HC participants (*p*=.019, *p*=.015, *p*=.030 respectively). Also, IBS patients were more alexithymic (TAS-20 without items 16 and 20: *p*=.035; TAS-20: *p*=.052) and tended to have more DIF (*p*=.061) than HC participants, and had more DIA than HC participants (*p* < .001) and UC patients (*p*=.004). In addition, there was a significant effect of the disease on the cortisol levels [*Welch F*(3,43.90)=4.45, *p*=.008]. UC patients had the lowest cortisol levels compared to CD patients (*p*=.032) (see details in **Table 1**).

**Table 1:** Socio-demographic, medical, and psychological data.

	HC (n=26)	IBS (n=24)	UC (n=18)	CD (n=21)	Group comparisons
Mean age (years)	36.23 ± 10.07	38.21 ± 11.26	40.94 ± 10.78	40.29 ± 11.17	_
Visceral pain (/10)	0.31 ± 1.19	2.25 ± 2.31^***^	0.94 ± 1.55	1.29 ± 1.62	IBS > HC
Anxiety (/80)	31.38 ± 7.62	41.5 ± 10.8^***^	33.5 ± 6.10	39.26 ± 10.03^*^	IBS;CD > HC
Depression (/60)	8.88 ± 4.48^***^	19.63 ± 9.37	10.17 ± 7.01^***^	13.67 ± 6.09^*^	IBS > HC;UC;CD
Alexithymia (TAS-20 without items 16 and 20)	30.12 ± 6.06	36.19 ± 9^*^	34.56 ± 8.5	36.75 ± 5.12^*^	IBS;CD > HC
DAF (/32)	12.96 ± 3.61	15.78 ± 5.68	14.59 ± 4.58	16.86 ± 3.32^*^	CD > HC
DIA (/8)	2.5 ± 1.03	4.82 ± 2.08^***^	3.06 ± 1.21^**^	3.95 ± 1.76^*^	IBS;CD > HC IBS > UC
EOT without items 15,16,20 (/20)	9.27 ± 2.51	9.64 ± 1.99	10.29 ± 2.91	9.71 ± 2.45	_
PAS (/20)	9.04 ± 2.3	10.23 ± 3.75	11.18 ± 3.11	11.33 ± 2.99	_
Alexithymia (TAS-20)	34.04 ± 6.51	40.20 ± 9.11^t^	39.00 ± 9.40	40.40 ± 5.37^*^	IBS;CD > HC
DIF (/28)	10.19 ± 3.24	13.33 ± 5.85 ^t^	10.94 ± 3.46	13.76 ± 3.60^*^	IBS;CD > HC
DF (/20)	9.15 ± 2.46	9.92 ± 4.14	10.33 ± 3.38	11.57 ± 2.82	_
EOT (/32)	14.88 ± 3.82	14.75 ± 4.08	17.22 ± 4.29	15.57 ± 3.41	_
HFnu	44.32 ± 17.72	42.74 ± 17.01	37.74 ± 17.82	33.98 ± 19.86	_
Cortisol (nmol/l)	369.04 ± 143.23	338.38 ± 184.36	251.11 ± 103.76^*^	445.55 ± 337.46	CD > UC

***≤.001; ^**^≤.01; ^*^≤.05; ^t^ ≤.07. Data are expressed as means ± SD. TAS-20, Toronto Alexithymia Scale; DAF, Difficulty in awareness of feelings; EOT, Externally oriented thinking; DIA, Difficulty in interoceptive abilities dimension; PAS, Poor affective sharing; DIF, Difficulty identifying feelings; DDF, Difficulty describing feelings; HFnu, High Frequency Normalized unit.

Contribution of DAF and DIA dimensions in predicting the presence of IBS. When entering anxiety in addition to depression as a predictor of the presence of IBS into Step 1, the full regression equation was significant, *χ^2^*(2)=22.45, *p* < .001; *R^2^ Nagelkerke*=.51. The model correctly classified 85.1% of cases. Only depression was a significant predictor of the presence of IBS, *W(1)*=8.01, *p*=.004. The introduction of the HFnu index and cortisol levels in the model (Step 2) also led to a significant equation, *χ^2^*(4)=22.52, *p* < .001; *R^2^ Nagelkerke*=.51. However, the introduction of physiological parameters did not add a significant explanation for the presence of IBS (Δ*χ^2^*(2)=.07, *p*=.968) contrary to the introduction of the interest variables, DAF and DIA dimensions in Step 3 (Δ*χ^2^*(2)=10.94, *p*=.004). At this last step, the full regression equation was significant (*χ^2^*(6)=33.46, *p* < .001; *R^2^ Nagelkerke*=.68) and correctly classified 87.2% of cases. Only DIA was a significant predictor of the presence of IBS, *W(1)*=6.27, *p*=.01. DAF and depression were not significant, as evidenced by confidence intervals including zero. This model was better than the first model [Δ*χ^2^*(4)=11.01, *p*=.026]. In summary, in the best model, only DIA factor was a significant predictor of IBS presence. Results are reported in **Table 2**.

**Table 2:** Summary of hierarchical logistical regression analysis predicting the presence of irritable bowel syndrome (IBS) (95% BCa bootstrap confidence intervals based on 5,000 samples).

	b	p	95% Confidence Interval		95% Confidence Interval for Odds Ratio		
			Lower	Upper	Lower	Odds	Upper
*Step 1*							
Constant	−4.48	.003^**^	−11.38	−1.88			
Anxiety	0.05	.225	−0.03	0.23	0.96	1.05	1.15
Depression	0.19	.004^**^	0.06	0.46	1.06	1.21	1.37
*Step 2*							
Constant	−4.53	.006^**^	−16.71	−0.92			
Anxiety	0.05	.243	−0.03	0.41	0.96	1.05	1.16
Depression	0.19	.002^**^	0.07	0.75	1.06	1.21	1.38
HFnu	−0.004	.860	−0.12	0.09	0.95	1	1.04
Cortisol	<0.001	.906	−0.02	0.01	1	1	1.01
*Step 3*							
Constant	−2.43	.223	−401.51	646.37			
Anxiety	0.05	.258	−2.43	15.01	0.94	1.05	1.18
Depression	0.24	.024	−0.06	80.25	1.03	1.28	1.58
HFnu	−0.04	.327	−21.73	2.17	0.90	0.96	1.03
Cortisol	0.001	.492	−0.53	0.43	1	1	1.01
DAF	−0.42	.033	−146.99	0.23	0.42	0.65	1.01
DIA	1.25	.010^**^	0.35	367.91	1.31	3.48	9.21

^**^≤.01. HFnu, High Frequency Normalized unit; DAF, Difficulty in awareness of feelings; DIA, Difficulty in interoceptive abilities dimension of the Toronto Alexithymia Scale; DIF, Difficulty identifying feelings; DDF, Difficulty describing feelings.

Contribution of DAF and DIA dimensions in predicting the presence of UC. When entering anxiety in addition to depression as a predictor of UC, the result of the full regression equation was not significant, *χ^2^*(2)=1.07, *p*=.587; *R^2^ Nagelkerke*=.03. However, at Step 2, when introducing HFnu index and cortisol levels in the model, the full regression equation was significant [*χ^2^*(4)=9.81, *p*=.044; *R^2^ Nagelkerke*=.28] and correctly classified 71.4% of cases. The difference from Step 1 revealed that the introduction of physiological parameters added an explanation of the prediction of the presence of UC, Δ*χ^2^*(2)=8.74, *p*=.013. However, only cortisol levels were a significant predictor of UC, *W(1)*=4.67, *p*=.035. At Step 3, the introduction of the interest variables, DAF and DIA dimensions, did not improve the model. Model 3 explained less well the presence of UC than model 2 [Δ*χ^2^*(2)=0.04, *p*=.982] and the full regression model was not significant, *χ^2^*(6)=9.85, *p*=.131; *R^2^ Nagelkerke*=.28. In summary, in the best model (Step 2), only cortisol levels were a significant predictor of UC presence. Results are reported in **Table 3**.

**Table 3:** Summary of hierarchical logistical regression analysis predicting the presence of ulcerative colitis (UC) (95% BCa bootstrap confidence intervals based on 5,000 samples).

	b	p	95% Confidence Interval		95% Confidence Interval for Odds Ratio		
			Lower	Upper	Lower	Odds	Upper
*Step 1*							
Constant	−1.64	.309	−6.60	1.45			
Anxiety	0.03	.621	−0.07	0.19	0.94	1.03	1.13
Depression	0.04	.472	−0.12	0.19	0.93	1.04	1.17
*Step 2*							
Constant	2.04	.384	−3.64	12.01			
Anxiety	0.04	.52	−0.1	0.24	0.93	1.04	1.15
Depression	0.004	.961	−0.24	0.25	0.88	1	1.15
HFnu	−0.02	.315	−0.11	0.03	0.94	0.98	1.02
Cortisol	−0.01	.035^*^	−0.04	−0.002	0.98	0.99	1
*Step 3*							
Constant	2.36	.469	−8.13	18.58			
Anxiety	0.04	.515	−0.15	0.35	0.93	1.04	1.16
Depression	0.01	.906	−0.50	0.33	0.86	1.01	1.19
HFnu	−0.02	.361	−0.15	0.06	0.93	0.98	1.02
Cortisol	−0.01	.029^*^	−0.07	−0.002	0.98	0.99	1
DAF	−0.02	.869	−0.54	0.82	0.76	0.98	1.26
DIA	0.01	.968	−1.31	2.17	0.47	1.01	2.19

^*^≤.05. HFnu, High Frequency Normalized unit; DAF, Difficulty in awareness of feelings; DIA, Difficulty in interoceptive abilities dimension of the Toronto Alexithymia Scale; DIF, Difficulty identifying feelings; DDF, Difficulty describing feelings.

Contribution of DAF and DIA dimensions in predicting the presence of CD. When entering anxiety in addition to depression as a predictor of CD, the full regression equation was significant, *χ^2^*(2)=11.21, *p*=.004; *R^2^ Nagelkerke*=.30. The model correctly classified 68.9% of cases. Only depression was a predictor of CD, *W(1)*=3.51, *p*=.027. The introduction of the HFnu index and cortisol levels in the model did not add an explanation (Δ*χ^2^*(2)=4.35, *p*=.113) despite the fact that the full regression equation was significant, *χ^2^*(4)=15.56, *p*=.004; *R^2^ Nagelkerke*=.39. Finally, the introduction of the interest variables, DAF and DIA dimensions, improved the model, *χ^2^*(6)=25.67, *p* < .001; *R^2^ Nagelkerke*=.58. Model 3 better explained the presence of CD than model 2 (Δ*χ^2^*(2)=10.10, *p*=.006) or model 1 (Δ*χ^2^*(4)= 14.46, *p*=.006), and correctly classified 80% of cases. However, neither the psychological nor the physiological parameters predicted the presence of CD. Although anxiety and DIA tended to be significant in the model, the confidence intervals included zero, revealing unreliable results. In summary, the best model was Step 3 and no factor was a significant predictor of CD, even if the introduction of DIA and DAF factors significantly improved the model. Results are reported in **Table 4**.

**Table 4:** Summary of hierarchical logistical regression analysis the presence of Crohn’s disease (CD) (95% BCa bootstrap confidence intervals based on 5,000 samples).

	b	p	95% Confidence Interval		95% Confidence Interval for Odds Ratio		
			Lower	Upper	Lower	Odds	Upper
*Step 1*							
Constant	−4.29	.001^***^	−9.20	−1.76			
Anxiety	.07	.072	−0.01	.23	0.99	1.08	1.18
Depression	.13	.027^*^	0.02	.35	0.99	1.14	1.31
*Step 2*							
Constant	−4.23	.008^**^	−14.31	−1.15			
Anxiety	.09	.028	0.001	0.37	0.99	1.09	1.20
Depression	.15	.066	−0.02	0.61	0.99	1.16	1.36
HFnu	−.04	.071	−0.19	−0.001	0.92	0.96	1
Cortisol	.002	.408	−0.004	0.01	1	1	1.01
*Step 3*							
Constant	−9.52	.001^***^	−1365.01	−4.51			
Anxiety	.11	.051	−0.26	19.76	0.98	1.11	1.26
Depression	.06	.553	−6.65	22.64	0.87	1.06	1.29
HFnu	−.05	.074	−12.58	0.002	0.90	0.95	1
Cortisol	.01	.077	−0.01	0.95	1	1.01	1.01
DAF	.20	.260	−5.1	18.93	0.88	1.22	1.68
DIA	.73	.058	−0.71	170.57	0.97	2.08	4.49

***≤.001; ^**^≤.01; ^*^≤.05. HFnu, High Frequency Normalized unit; DAF, Difficulty in awareness of feelings; DIA, Difficulty in interoceptive abilities dimension of the Toronto Alexithymia Scale.

### Additional Analysis

Despite the fact that the DIA dimension had good internal reliability, this dimension includes only two items (57). For this reason, we have carried out additional analyses by including, instead of the DAF and DIA dimensions, the theoretical DIF and DDF dimensions of the TAS-20 as predictors. We decided to keep only these two dimensions because they included all the items distributed in the DAF and DIA dimensions.

Contribution of DDF and DIF dimensions in predicting the presence of IBS. When entering anxiety in addition to depression as a predictor of IBS, the full regression equation was significant, *χ^2^*(2)=23.10, *p* < .001; *R^2^ Nagelkerke*=.50. The model correctly classified 83.7% of cases. Only depression was a predictor of IBS, *W(1)*=7.71, *p*=.005. The introduction of HFnu index and cortisol levels in the model did not add an explanation (Δ*χ^2^*(2)=0.34, *p*=.844) despite the fact that the full regression equation was significant, *χ^2^*(4)=23.44, *p* < .001; *R^2^ Nagelkerke*=.51. Finally, the introduction of the interest variables, the DDF and DIF dimensions, did not improve model, Δ*χ^2^*(2)=.04, *p*=.98, *χ^2^*(6)=23.48, *p*=.001; *R^2^ Nagelkerke*=.51. Model 1 better explained the presence of IBS than model 3 (Δ*χ^2^*(4)=.38, *p*=.984). In summary, the best model was Step 1 and only depression scores were a significant predictor of IBS. Results are reported in **Table 5**.

**Table 5:** Summary of hierarchical logistical regression analysis predicting the presence of irritable bowel syndrome (IBS) (95% BCa bootstrap confidence intervals based on 5,000 samples).

	b	p	95% Confidence Interval		95% Confidence Interval for Odds Ratio		
			Lower	Upper	Lower	Odds	Upper
*Step 1*							
Constant	−4.35	.003^**^	−11.08	−1.80			
Anxiety	0.053	.226	−0.03	0.23	0.96	1.05	1.16
Depression	0.18	.005^**^	0.07	0.42	1.05	1.20	1.36
*Step 2*							
Constant	−4.44	.006^**^	−14.70	−0.97			
Anxiety	0.06	.223	−0.03	0.36	0.96	1.06	1.16
Depression	0.19	.003^**^	0.07	0.61	1.05	1.20	1.37
HFnu	−0.01	.713	−0.12	0.06	0.95	0.99	1.04
Cortisol	0.001	.778	−0.01	0.01	1	1	1.01
*Step 3*							
Constant	−4.74	.055^t^	−291.88	6.43			
Anxiety	0.06	.260	−.09	7.20	0.96	1.06	1.17
Depression	0.18	.014^*^	.04	29.74	1.02	1.19	1.4
HFnu	−0.01	.726	−3.82	.12	0.95	0.99	1.04
Cortisol	0.001	.737	−.23	.02	1	1	1.01
DIF	0.02	.858	−5.35	3.23	0.79	1.02	1.31
DDF	0.01	.897	−18.69	2.01	0.75	1.01	1.36

^**^≤.01; ^*^≤.05; ^t^ ≤.07. HFnu, High Frequency Normalized unit; DIF, Difficulty identifying feelings; DDF, Difficulty describing feelings on the Toronto Alexithymia Scale.

Contribution of DDF and DIF dimensions in predicting the presence of UC. When entering anxiety in addition to depression as a predictor of UC, the result of the full regression equation was not significant, *χ^2^*(2)=0.98, *p*=.613; *R^2^ Nagelkerke*=.03. However, at Step 2, when introducing HFnu index and cortisol levels in the model, the full regression equation was significant (*χ^2^*(4)=10.87, *p*=.028; *R^2^ Nagelkerke*=.30) and correctly classified 72.1% of cases. The difference from Step 1 revealed that the introduction of physiological parameters added an explanation of the prediction of the presence of UC, Δ*χ^2^*(2)=9.89, *p*=.007. However, only cortisol levels were a significant predictor of UC, *W(1)*=5.11, *p*=.026. At Step 3, the introduction of the interest variables, the DDF and DIF dimensions, did not improve the model (Δ*χ^2^*(2)=2.14, *p*=.344), but the full regression model was significant, *χ^2^*(6)=13, *p*=.043; *R^2^ Nagelkerke*=.35. Therefore, the best model was Step 2 and only cortisol levels were a significant predictor of UC. Results are reported in **Table 6**.

**Table 6:** Summary of hierarchical logistical regression analysis predicting the presence of ulcerative colitis (UC) (95% BCa bootstrap confidence intervals based on 5,000 samples).

	b	p	95% Confidence Interval		95% Confidence Interval for Odds Ratio		
			Lower	Upper	Lower	Odds	Upper
			Lower	Upper	Lower	Odds	Upper
*Step 1*							
Constant	−1.67	.308	−6.66	1.57			
Anxiety	.03	.534	−0.07	0.20	0.94	1.03	1.13
Depression	.03	.583	−0.15	0.17	0.92	1.03	1.16
*Step 2*							
Constant	2.22	.347	−3.30	13.39			
Anxiety	.04	.471	−0.10	0.25	0.93	1.04	1.16
Depression	−.004	.959	−0.24	0.22	0.87	1	1.14
HFnu	−.02	0.3	−0.11	0.03	0.94	0.98	1.02
Cortisol	−.01	.026^*^	−0.04	−0.003	0.98	0.99	1
*Step 3*							
Constant	2.88	.431	−12.37	19.99			
Anxiety	.07	.218	−0.10	0.66	0.95	1.08	1.21
Depression	.09	.442	−0.51	0.59	0.89	1.09	1.33
HFnu	−.04	.147	−0.21	0.06	0.91	0.96	1.01
Cortisol	−.01	.011^*^	−0.11	−0.003	0.98	0.99	1
DIF	−.27	.148	−1.57	0.36	0.51	0.76	1.14
DDF	.12	.514	−0.37	1.76	0.84	1.12	1.51

^*^≤.05. HFnu, High Frequency Normalized unit; DIF, Difficulty identifying feelings; DDF, Difficulty describing feelings on the Toronto Alexithymia Scale.

Contribution of DDF and DIF dimensions in predicting the presence of CD. When entering anxiety in addition to depression as a predictor of CD, the full regression equation was significant, *χ^2^*(2)=11.21, *p*=.004; *R^2^ Nagelkerke*=.30. The model correctly classified 68.9% of cases. Only depression was a predictor of the presence of CD, *W(1)*=3.51, *p*=.025. The introduction of the HFnu index and cortisol levels in the model did not add an explanation (Δ*χ^2^*(2)=4.35, *p*=.113) despite the fact that the full regression equation was significant, *χ^2^*(4)=15.56, *p*=.004; *R^2^ Nagelkerke*=.39. Finally, the introduction of the interest variables, the DDF and DIF dimensions, improved the model, *χ^2^*(6)=28.19, *p* < .001; *R^2^ Nagelkerke*=.62. Model 3 better explained the presence of CD than model 2 (Δ*χ^2^*(2)=12.62, *p*=.002) or model 1 (Δ*χ^2^*(4)= 16.98, *p*=.002), and correctly classified 82.2% of cases. Only DDF predicted CD, *W(1)*=6.16, *p* < .001. Although anxiety and cortisol levels tended to be significant in the model, the confidence intervals included zero, revealing unreliable results. In summary, the best model was Step 3 and only DDF was a significant predictor of CD. Results are reported in **Table 7**.

**Table 7:** Summary of hierarchical logistical regression analysis predicting the presence of Crohn’s disease (CD) (95% BCa bootstrap confidence intervals based on 5,000 samples).

	b	p	95% Confidence Interval		95% Confidence Interval for Odds Ratio		
			Lower	Upper	Lower	Odds	Upper
*Step 1*							
Constant	−4.29	.002^**^	−9.38	−1.87			
Anxiety	.08	.076	−0.01	0.23	0.99	1.08	1.18
Depression	.13	.025^*^	0.01	0.35	0.99	1.14	1.31
*Step 2*							
Constant	−4.23	.008^**^	−14.87	−1.02			
Anxiety	.09	.033^*^	.001	.38	0.99	1.09	1.2
Depression	.15	.067	−.01	.61	0.99	1.16	1.36
HFnu	−.04	.074	−.17	.001	0.92	0.96	1
Cortisol	.002	.401	−.004	.01	1	1	1.01
*Step 3*							
Constant	−15.13	.001^***^	−1605.15	−8.86			
Anxiety	.16	.015	−.10	18.32	1.01	1.17	1.36
Depression	.11	.331	−1.19	23.91	0.87	1.11	1.43
HFnu	−.03	.193	−5.14	.07	0.92	0.97	1.01
Cortisol	.01	.016	−.003	.61	1	1.01	1.01
DIF	.13	.438	−1.12	25.65	0.8	1.14	1.63
DDF	.56	< .001^***^	.15	53.73	1.12	1.74	2.71

^***^≤.001; ^**^≤.01; ^*^≤.05. HFnu, High Frequency Normalized unit; DIF, Difficulty identifying feelings; DDF, Difficulty describing feelings on the Toronto Alexithymia Scale.

## Discussion

The aim of this paper was to explore the contribution of DIA and DAF in predicting the presence of gastrointestinal pathologies which could be considered vulnerabilities. After checking for the effects of psychological (i.e., anxiety, depression) and physiological (i.e., ANS and HPA axis activities at rest) factors, the results showed that DIA was a significant predictor only for IBS patients. In IBD patients, despite the fact that UC and CD are usually grouped in the same category (i.e., IBD), the factors predicting the presence of these pathologies are different. UC was predicted by biological parameters (i.e., cortisol level), while CD was not predicted by any factors, although some psychological factors (i.e., depression and DIA scores) tended to explain the presence of this disease. Analyses with the original TAS-20 dimensions (i.e., DIF and DDF) showed that the lack of consideration of interoceptive abilities (i.e., DIA dimension) in IBS or IBD patients led to different predictions. Indeed, in IBS patients, depression scores were a significant predictor of the presence of the pathology, while in CD patients it was the DDF scores. Cortisol remained the predictor of the presence of UC disease.

In this study, IBS patients exhibited an alteration of interoceptive abilities that might reflect increased attention to their visceral sensations and an intense concern for their own health (26). All internal stimuli, including visceral sensations, are monitored by interoceptive mechanisms, closely associated with pain (66). An alteration in interoceptive abilities could thus explain the high levels of visceral pain as observed in the IBS group, but also could explain the presence of high levels of alexithymia. Indeed, interoceptive abilities are difficult to separate from the ability to identify and describe feelings. Being aware of one’s internal bodily changes necessarily implies being able to identify them. As a consequence, poor discrimination and sensitivity of body states would lead to difficulties in regulating one’s own emotional states in an adaptive way, and thus to the use of more somatic patterns to describe emotions (67, 68), which can, in turn, contribute to the development of psychological and physical disorders. In addition, interoceptive dysfunctions are associated with psychopathology (69) such as panic disorders, anxiety syndromes, depression, schizophrenia (34), addictions, eating disorders and somatic symptom disorders (70). One of the major components of interoception is the immune communication between the periphery and the brain. Through various channels of communication, including the interoceptive pathway, the peripheral inflammatory state is transmitted to the brain. These signals are then integrated and translated into experiential feeling states and can lead to changes in reward/punishment motivational behaviors, mood, cognition, and visceral pain perception. When inflammation is severe or chronic, these changes may be comparable to those observed in major depressive disorders (71). Consistent with this theory, a review of the literature mentions a link between inflammation and depression and suicidal behaviors (72). Similarly, high levels of C-reactive protein in schizophrenia suggest a role of inflammation in the pathogenesis of this psychopathology (73). The potential roles of interoception and inflammation in the aetiology and/or symptomatology of a number of psychiatric conditions makes understanding these relationships a central goal for clinical psychology. Understanding these relationships could be instructive about how the brain perceives the body and could help identify the mechanisms by which interoception and inflammation lead to a risk of developing or relapsing into pathology. Therefore, DIA could be the aetiology of a greater vulnerability to develop or relapse into IBS when faced with stressful situations. This study could provide new targets for effective treatment.

Since pharmacotherapy has limited effects on IBS (74), physicians are increasingly using psychological interventions such as cognitive behavioral therapies (CBT) or interpersonal psychotherapy (IPT), (75) and hypnotherapy (76). Among these therapies, CBT seems to be the most effective practice for alleviating IBS symptoms compared to hypnosis, psychodynamics and relaxation (77). This practice involves a combination of different techniques (i.e., psycho-education, relaxation strategies, cognitive restructuring, problem-solving techniques or coping skills, interoceptive, and situational exposure techniques) (78). Its aim is to modify dysfunctional behaviors and thoughts, but also to promote awareness of the body’s sensations as felt in the gastrointestinal tract and during breathing. In particular, the integration of interoceptive exposure exercises in CBT has shown their effectiveness in comparison to stress management intervention and an attention control group (79). Therefore, Craske and colleagues (79) and Kinsinger (78) showed the need to focus on interoceptive abilities when treating IBS patients. IBS patients present a hypersensitivity to and a hypervigilance of gastrointestinal symptoms that may contribute to visceral anxiety. In addition, IBS patients avoid many situations (e.g., situations without access to restrooms) and adopt safe behaviors (e.g., taking medication during a trip) which also contributes to maintaining symptom-related anxiety. The incorporation of interoceptive exposure in a CBT programme would thus reduce visceral anxiety by acting on the fear of gastrointestinal sensations. The practice of these exposure exercises could lead to increased self-efficacy and change the misperception about the nociception of gastrointestinal sensations and the urgency of symptoms. The results of this study therefore support the need to improve the interoceptive capacities of patients with IBS for alleviating symptoms and ideally preventing relapses. New longitudinal studies in which the intervention’s target is the improvement of interoceptive abilities must therefore be conducted.

For UC patients in remission, low cortisol levels predicted the presence of this pathology. Cortisol has an antiinflammatory action and reduces visceral pain. Low cortisol levels are associated with an increase in pain perception (80) and may reflect dysfunction of the HPA axis (81). This axis inhibits the sympathetic (adrenergic) nervous system (82, 83). An alteration of these feedback loops could favor overactivity of the sympathetic axis during rest and when faced with a stressful situation. It therefore makes UC patients more vulnerable to stress. In addition, low cortisol levels could reflect habituation to an environment considered as hostile (84, 85). This could result in the body’s inability to maintain a state of homeostasis. In the long term, this could lead to the development of many pathologies related to chronic stress (86, 87), or to a greater vulnerability to relapse into the disease. Another explanation is that low cortisol levels could reflect a previous trauma in early life. Repeated and prolonged exposure to one or more adverse situations in childhood (e.g., physical trauma, loss of a parent, physical/sexual abuse) increases an individual’s susceptibility to somatic and psychiatric diseases (88). An explanation of this link is based on brain-gut-microbiota axis, involving the ANS, the HPA axis, and the gastrointestinal tract (89). In a review article, Agorastos and colleagues detail the various mechanisms involved in early-life stress and trauma (90). They mentioned that early-life trauma is associated with insufficient glucocorticoid signalling that may have negative effects on the regulation of the immune system and ANS functioning. In addition, a repeated activation of the stress system may result in increased or decreased peripheral cortisol levels due to an alteration of the HPA axis. The HPA axis is closely interconnected with the ANS. An alteration of the HPA axis may deregulate the central autonomic network, thereby altering peripheral ANS activity and overall stress responsiveness. This can then lead to a dysregulation of inflammatory feedback mechanisms, which promotes the development of inflammatory-related or immunosuppressed medical conditions. Even years later (91, 92), these alterations may persist. Thus, this assumption of repeated or chronic stress in early life in UC patients would not be surprising given that the prevalence of early adversities (i.e., exposure to parental domestic violence, childhood physical abuse, childhood sexual abuse) is high in these patients (93).

Despite the inclusion of DIA and DAF assessment in the best model, no factors predicted CD. A 1-year longitudinal study found that psychosocial factors associated with chronic disease (i.e., stress induced by the physical symptoms of the disease, the use of emotion-focused coping, loss of resources and the quality of life related to chronic diseases) were the best predictors of vulnerability to relapse in CD (94). Due to their longitudinal dimension, the quantitative and qualitative data in this paper provided a comprehensive assessment of the stressors and quality of life associated with relapse. However, psychosocial stressors act through complex neuroendocrine immune pathways. Thus, biological factors underpinned psychosocial factors when considering a relapse in CD. A study integrating physiological and psychological factors revealed that physiological factors were independent predictors of relapse (higher CRP, fistulizing behavior, and colon-confined disease) and psychological factors were protectors of relapse (patients living in low stress conditions and with low avoidance rates) (95). Moreover, among CD patients, some have an overlap between their symptoms and IBS symptoms (96). CD patients suffering IBS-like symptoms present visceral hypersensitivity (97) while patients with CD alone have instead visceral hyposensitivity (64). Here, we do not differentiate between CD with IBS-like symptoms and CD-like symptoms. It may be that the DIA factor can predict the presence of CD with concomitant features of IBS. Further studies should be carried out to further develop this hypothesis.

For IBD patients, treatment typically focuses on the prevention of disease symptoms and inflammatory episodes, addressed through medications and/or surgery (2, 98). Yet, a White Paper from the American Gastroenterological Association provided evidence-based recommendations from health centres for the integration of psychosocial management in IBD care (99). Moreover, the results of this study and the literature highlight the importance of considering psychological factors in the vulnerability of IBD to develop chronic stress, which could lead to the recurrence or aggravation of gastrointestinal pathologies. However, the integration of psychosocial care seems to be more important for CD than UC patients. However, studies on the effectiveness of CBT in the treatment of HPA axis dysfunction in UC have shown that this practice increases cortisol levels by reversing some of the effects induced by low physical activity levels, depression and stress in early life (81). CBT may therefore influence the management of UC. However, recent research has reported better outcomes for CD patients than UC patients after CBT therapy, but these data were self-reported, were not from a standardized measure, and thus have to be interpreted with caution (100).

In Fournier and colleagues (57) and in this study, the DIA dimension had good internal reliability. However, this dimension includes only two items. For this reason, we carried out additional analyses by including the theoretical DIF and DDF dimensions of the TAS-20 as predictors. For UC patients, the introduction of the DDF and DIF dimensions instead of the DIA and DAF dimensions did not change the results. The best model was the model at Step 2 and only cortisol levels predicted the presence of an UC. However, for IBS patients, the best model became the one at Step 1 and only depression scores could predict the presence of this disorder. Without considering the DIA and DAF dimensions, we would have made wrong conclusions about the targets of action. Indeed, we could have concluded that the target of action must be depression when in fact it would be interoception involving other types of therapeutic interventions. Regarding CD patients, the best model reported that DDF scores predicted the presence of this disease. Yet, in the first analysis, the DAF dimension, that included items from the DDF (item 2, 4, 11) and DIF (item 1, 6, 9, 13, 14) dimensions, was not a significant predictor of CD. This suggests that the main difficulty of CD patients is in describing feelings to others and not a global difficulty in awareness of feelings. CD patients could be able to identify their feelings but might be unable to find the right words to describe them to others. CD patients would have more semantic difficulties (revealed by the original structure of the TAS) while IBS patients would have more visceral difficulties (revealed by items 3 and 7 of the new TAS structure). Thus, both forms of the TAS (traditional and new structures) are interesting because they reveal the weak point of each of the pathologies with regard to the identification and description of one’s body sensations or feelings. The new factorial structure can be helpful for predicting some diseases like IBS, but not for predicting others like CD where DDF is the strongest predictor. Therefore, the traditional factor structure is still relevant for predicting diseases other than IBS. However, for IBS, there is a need to use the new subscales of alexithymia. These data lead us to believe that it would be useful to keep, in alexithymia assessment, a dimension that allows the evaluation of the difficulty in describing feelings to others, but also to include a new dimension of interoception based on a factor for which new items need to be developed.

This study had various limitations. First, the number of participants included in the study was small and the number of predictors high. However, all validity criteria for statistical analyses were verified and met, making the results reliable. Second, patients with IBS were diagnosed from Rome II criteria (52). Now, we are in Rome IV criteria (101). The main differences between these two criteria concern the frequency and duration of symptoms (at least 12 weeks out of the preceding 12 months for the Rome II criteria and at least 1 day/week in the last 3 months for the Rome IV criteria), the addition of a classification of IBS by subtypes based on predominant bowel habits on days with abnormal bowel movements, and the term “discomfort” was removed from the current definition and diagnostic criteria to keep only the notion of abdominal pain. Another significant change was that the symptom of bloating as a primary symptom was eliminated from the definition. It is therefore possible that some IBS patients may have been misdiagnosed. Third, the DIA measurement was obtained from only two items of the TAS-20. Despite good internal consistency for this dimension, it is necessary to develop a new alexithymia scale that would contain an interoceptive dimension. Current studies highlight the importance of considering interoceptive abilities when studying alexithymia and the results of this study support the interest of including this dimension in alexithymic individuals with chronic pathologies. In addition, it would be interesting to conduct new studies with validated interoceptive measures such as the Multidimensional Assessment of Interoceptive Consciousness (MAIA) (102) to measure subjective interoceptive abilities, and a heartbeat-counting task to measure objective interoceptive abilities, different from Schandry’s task (103) which seems controversial (104, 105). Fourth, our study does not consider all the alterations in the biopsychosocial sphere mentioned in the literature such as inflammatory markers. It may be interesting to conduct another study that takes more predictors into consideration.

In summary, this paper highlights the importance of considering interoceptive abilities in gastrointestinal disorders as a vulnerability factor when patients are faced with stress situations, especially in IBS patients. In the IBS group, the DIA dimension was predictive of the presence of IBS, suggesting that when faced with a new stressful situation, patients in remission would have difficulty in perceiving their own internal body changes and thus would have difficulty properly regulating their emotions. Faced with intense and/or repetitive stress, this could contribute to a relapse into the disease. In the CD group, difficulties describing feelings to others would be a predictor of the presence of this disorder. In light of the literature and our results, the contribution of factors such as the DIA and DDF dimensions and cortisol levels could be significant. Further studies are needed to deepen or improve knowledge. In the UC group, predictors seem to belong more to the physiological sphere. Even if CD and UC are grouped under the term “IBD,” it seems that their psychophysiological functioning is completely different. Genetic susceptibility, environmental factors, and altered gut microbiota, leading to dysregulated innate and adaptive immune responses, are the core issue of IBD (106, 107). Despite some common aetiological factors between UC and CD, some differences exist at the macroscopic level since the digestive expression of the disease restricted to the rectum and colon in UC is generalised to the all tract in the CD, but also at the autonomic level and psychologic level. For instance, the autonomic balance between UC and CD has been shown to be different depending on affects and psychological adjustment (18). Moreover CD patients generally report more anxiety and depression than UC patients (108, 109). However, few studies compare the functioning of the HPA axis between UC and CD, which does not allow us to conclude on the role of cortisol in UC. It is therefore of great importance to distinguish UC from CD when studying IBD. The model presented in this paper must now be tested under experimental conditions in patients with regards to relapse. These results are promising and open new perspectives for the healthcare of patients with gastrointestinal diseases, such as CBT.

## Data Availability Statement

The datasets generated for this study are available on request to the corresponding authors.

## Ethics Statement

The studies involving human participants were reviewed and approved by the Ethical Committee of the CHU (ref: CPP 08-CHUG-23; ClinicalTrials.gov Identifier: NCT010950421). The patients/participants provided their written informed consent to participate in this study.

## Author Contributions

SP, CD, FC, LM, and BB designed the study. BB and NM recruited the patients. SP and BB raised funds to finance the protocol. SP recruited the healthy controls for inclusion. SP and CD performed the experiments and collected psychological and physiological data. AG performed the biochemical assays. AF analysed the data. OL and AF interpreted the data. AF, OL, LM, and SP drafted the manuscript. FC, NM, AG, CD, and BB undertook a critical revision of the manuscript. All the authors approved the final version submitted for publication.

## Funding

This work was supported by the Association François Aupetit (AFA), the French National Society of Gastroenterology (SNFGE), and the Direction de la Recherche Clinique (DRC 2008 – BONAZ B.) of Grenoble University Hospital. The funders had no role in study design, data collection and analysis, decision to publish or preparation of the manuscript. All authors of this article had authority over manuscript preparation and the decision to submit the manuscript for publication.

## Conflict of Interest

The authors declare that the research was conducted in the absence of any commercial or financial relationships that could be construed as a potential conflict of interest.

## Footnotes

1 Item 3: *“I have physical sensations that even doctors don’t understand”.*

2 Item 7: *“I am often puzzled by sensations in my body”.*
